# Bluestone’s Emergency Department Early Response Program: Promoting High-Quality and Safe Transitions

**DOI:** 10.1093/geroni/igaf122.597

**Published:** 2025-12-31

**Authors:** Grace Wittenberg, Martha Etzell, Peter Serina, Nichole Stetten, Ann Reddy, Ellen McCreedy

**Affiliations:** Brown University, Providence, Rhode Island, United States; Bluestone Physician Services, Stillwater, Minnesota, United States; Salem Hospital, Salem, Massachusetts, United States; Brown University, Providence, Rhode Island, United States; Brown University, Providence, Rhode Island, United States; Brown University, School of Public Health, Providence, Rhode Island, United States

## Abstract

Almost half of all Assisted Living Community (ALC) residents transfer to the emergency department (ED) each year. For people living with dementia (PLWD), providing a medical history can be a challenge, resulting in prolonged stays and risk of delirium or hospital acquired infections. Bluestone Physician Services developed a ED early response program in which complex care managers (CCM) receive an electronic Admission, Discharge, and Transfer (ADT) notification when one of their patients registers at an ED. If the notification occurs during business hours, the CCMs call the ED using a script and follow-up with a structured fax. We conducted semi-structured interviews with 12 CCMs involved in the program to assess the feasibility and acceptability of the program. There were five themes: patients with dementia and those on hospice were especially likely to benefit from the program; strengths of the program, including increased communication between Bluestone and ED providers; weakness of the program, including a lack of awareness of the program among variable ED staff and challenges with timing the call and fax to maximize benefit. The CCMs also shared some learnings and adaptations that increased contact rate over time and individual success stories. In this session, we will also provide tips on how to integrate real-time ADT notifications into CCM workflows. Next steps include examining the ED provider, patient and caregiver perceptions of the program, quantifying the impact of the program on utilization, and analyzing how the program would perform in other health care settings.

